# THE IMPLICATIONS OF THE BUILD BACK BETTER BILL FOR AGING HEALTH POLICY

**DOI:** 10.1093/geroni/igac059.137

**Published:** 2022-12-20

**Authors:** Pamela Nadash

**Affiliations:** University of Massachusetts Boston, Boston, Massachusetts, United States

## Abstract

The Build Back Better Bill passed by the U.S. House of Representatives (HR 5376) included ambitious healthcare and long-term care provisions that would have greatly benefited older people, although the Bill itself represents a considerable scaling down of the Biden Administration’s original proposals. This presentation reviews key provisions of the Bill that would impact older people: paid family and medical leave, efforts to strengthen the direct care workforce, and investments in home and community-based services. In addition, the Bill expanded Medicare coverage to include hearing, dental, and vision coverage, and expanded tax credits for the medical expenses associated with family caregiving. Although the House bill failed to make it through the Senate intact, some of the provisions are likely to have staying power. This presentation reviews the Bill’s progress and assesses implications for future efforts to expand access to these benefits.

